# A metabolomic approach to identifying platinum resistance in ovarian cancer

**DOI:** 10.1186/s13048-015-0140-8

**Published:** 2015-03-26

**Authors:** Laila M Poisson, Adnan Munkarah, Hala Madi, Indrani Datta, Sharon Hensley-Alford, Calvin Tebbe, Thomas Buekers, Shailendra Giri, Ramandeep Rattan

**Affiliations:** Center for Bioinformatics, Henry Ford Hospital, Detroit, MI 48202 USA; Department of Public Health Sciences, Henry Ford Hospital, Detroit, MI 48202 USA; Josephine Ford Cancer Institute, Henry Ford Hospital, Detroit, MI 48202 USA; Division of Gynecology Oncology, Department of Women’s Health Services, Henry Ford Hospital, Detroit, MI 48202 USA; Department of Neurology, Henry Ford Health System, Detroit, MI 48202 USA

**Keywords:** Metabolomics, Ovarian cancer, Platinum resistance, A2780, C200, Methionine metabolism

## Abstract

**Background:**

Acquisition of metabolic alterations has been shown to be essential for the unremitting growth of cancer, yet the relation of such alterations to chemosensitivity has not been investigated. In the present study our aim was to identify the metabolic alterations that are specifically associated with platinum resistance in ovarian cancer. A global metabolic analysis of the A2780 platinum-sensitive and its platinum-resistant derivative C200 ovarian cancer cell line was performed utilizing ultra-high performance liquid chromatography/mass spectroscopy and gas chromatography/mass spectroscopy. Per-metabolite comparisons were made between cell lines and an interpretive analysis was carried out using the Kyoto Encyclopedia of Genes and Genomes (KEGG) metabolic library and the Ingenuity exogenous molecule library.

**Results:**

We observed 288 identified metabolites, of which 179 were found to be significantly different (*t*-test p < 0.05) between A2780 and C200 cells. Of these, 70 had increased and 109 had decreased levels in platinum resistant C200 cells. The top altered KEGG pathways based on number or impact of alterations involved the cysteine and methionine metabolism. An Ingenuity Pathway Analysis also revealed that the methionine degradation super-pathway and cysteine biosynthesis are the top two canonical pathways affected. The highest scoring network of altered metabolites was related to carbohydrate metabolism, energy production, and small molecule biochemistry. Compilation of KEGG analysis and the common network molecules revealed methionine and associated pathways of glutathione synthesis and polyamine biosynthesis to be most significantly altered.

**Conclusion:**

Our findings disclose that the chemoresistant C200 ovarian cancer cells have distinct metabolic alterations that may contribute to its platinum resistance. This distinct metabolic profile of platinum resistance is a first step towards biomarker development for the detection of chemoresistant disease and metabolism-based drug targets specific for chemoresistant tumors.

**Electronic supplementary material:**

The online version of this article (doi:10.1186/s13048-015-0140-8) contains supplementary material, which is available to authorized users.

## Background

Ovarian cancer is responsible for the highest mortality of all cancers of the female reproductive system. It accounts for approximately 3% of all cancers in women and is the fifth leading cause of cancer related death among women in the United States [1]. Ovarian cancers are generally sensitive to chemotherapy and often initially respond well to standard primary treatment with surgery and first-line platinum and taxane-based chemotherapy. However, approximately 70% of the patients experience disease relapse within 2 years of the initial treatment. Of these, only a few benefit from subsequent therapies using a platinum and taxane combination. Patients with a short time to disease progression and with no benefit to further platinum-based therapy are classified as having platinum-resistant disease, whereas those with long-lasting response to primary treatment and/or response to second-line platinum-based therapy are said to have platinum-sensitive disease. Even though the presence or development of platinum resistance is a major obstacle in successful ovarian cancer treatment, platinum therapy is still the principal treatment for recurrent tumors [2].

The antitumor activity of platinum has been shown to be due to the formation of intra-strand DNA adducts, which are irreparable and will eventually lead to cell apoptosis [3]. Additionally, cisplatin, a commonly used platinum-compound chemotherapy, is known to induce oxidative stress and endoplasmic reticulum stress, but the extent to which these pathways contribute to cell death is not yet established [4,5]. Platinum resistance has been attributed to reduced drug accumulation, improved drug efflux, drug inactivation, enhanced DNA repair ability and upregulation of anti-apoptotic or other survival genes [6,7]. While advancements have been made in understanding the molecular deregulation underlying chemoresistance, these have not translated into clinical applications to enhance the therapeutic outcome of platinum resistant tumors. Therefore, strategies addressing the identification of chemoresistant tumors that can be directly translated to clinic are required.

Metabolomics is a new discipline which evaluates diverse metabolite concentrations in biological specimens to gain insight into the ongoing metabolism. Metabolites are the end product of various metabolic pathways and may have application as biomarkers for cancer diagnosis, prognosis, and therapeutic evaluation [8]. Apart from revealing diagnostic and prognostic biomarkers, this profile of cell functioning at the metabolite level will help obtain an elementary understanding of the process of carcinogenesis and chemoresistance that may provide opportunities for early diagnosis and treatment.

Recent metabolomic based studies in ovarian cancer have been applied to the screening of urine, plasma, and tumor tissue from ovarian cancer patients and control populations [9-14]. These studies have endeavored to discriminate between healthy and ovarian cancer patients [9-11], profile malignant and borderline ovarian tumors [10], and detect recurrent tumors [12,14]. All of these studies clearly demonstrate that metabolomic profiles in the urine, plasma, or tumor tissue can distinctly separate healthy women from those with benign or malignant ovarian tumors, indicating that the science of metabolomics can be successfully applied for ovarian cancer characterization and identification.

Since all chemotherapeutic drugs are metabolically processed, it can be extrapolated that metabolism plays a vital role in chemoresponse of the tumors. Cell death whether by apoptosis or necrosis requires energy from the cell and involves regulation by various metabolic enzymes. Targeting of metabolic enzymes from key metabolic pathways, like glycolysis [15], fatty acid synthesis [16], and glucose transport [17], have been shown to enhance the cytotoxicity of various chemotherapeutic agents and radiotherapy. Moreover, cisplatin treatment has recently been shown to induce intracellular metabolic changes [18]. Thus, it can be postulated that chemoresistant tumor cells will have specific altered metabolism compared to chemosensitive tumor cells that could be detected by comparing their metabolites.

We designed our metabolomics-based study to identify metabolite variations that distinguish between platinum resistant and sensitive ovarian cancer cells. By using platinum sensitive A2780 and resistant C200 ovarian cancer cell lines, we are able to show that metabolite alterations can clearly separate the cells based on their platinum sensitivity. We identified significant metabolite variations in 6 different metabolic pathways participating in various signaling networks, with methionine metabolism and its associated metabolites being the centrally affected pathway.

## Methods

### Cell lines

A2780 and C200 cell lines were a kind gift from Dr. Thomas Hamilton, Fox Chase Cancer Center, PA. The cell lines were maintained in Roswell Park Memorial Institute media (HyClone-ThermoScientific; Waltham, MA) supplemented with 10% fetal bovine serum (BioAbChem; Ladson, SC) and insulin. For preparation of cells, cells were grown for 48 hours in insulin free media. Ten million cells were counted, washed with phosphate buffered saline and snap frozen.

### Metabolite assessment

Metabolomic profiling analysis was performed by Metabolon Inc. (Durham, NC) as previously described [19-22]. Briefly, sample preparation was conducted using an aqueous methanol extraction and the resulting extract were analyzed by ultra-performance liquid chromatography/mass spectroscopy (positive and negative modes) and gas chromatography/ mass spectroscopy. Raw data were extracted, peak-identified and quality control processed using Metabolon’s hardware and software. Compounds were identified by comparison to library entries of purified standards or recurrent unknown entities based on 3 criteria: retention index within a narrow retention index window of the proposed identification, nominal mass match to the library +/− 0.2 atomic mass units, and the mass spectroscopy/mass spectroscopy forward and reverse scores between the experimental data and authentic standards.

### Data analysis

To control for sample concentration, each metabolite intensity value was standardized as a ratio against the Bradford protein measure for that sample. Missing values which indicate a limit of detection by the mass spectrometer were replaced with a small value (one half the study minimum) for analysis. The data were visualized by plotting the first and second components of a partial least squares discriminant analysis (PLS-DA) model. A z-score plot was generated by plotting each metabolite intensity of the resistant C200 cells relative to the mean and standard deviation of the sensitive (A2780) cells. A one-unit change indicated a one standard deviation change in intensity away from the A2780 mean. Each observation represents 1 point on this z-score plot. The observations were organized by metabolites (rows) within super-pathway and sub-pathway. Metabolite intensities were compared between lines by a two-sample *t*-test per metabolite allowing for unequal variance on a log2 scale. Significant metabolites (at p < 0.01) were selected for inclusion in the heatmap. Metabolites (rows) were ordered first by super-pathway and then by direction of change. The columns (samples) are ordered by hierarchical clustering using Pearson correlation and complete linkage. Overrepresentation of changed molecules within super-pathway and sub-pathway was tested using a Fisher’s exact test per grouping. Change determined from the per-metabolite t-tests (p < 0.05) was classified according to direction of change from control, i.e., up, down, and unchanged.

### Pathway analysis

Pathway analysis was conducted in MetaboAnalyst (2.0; last accessed April 2013) using the Human Metabolon Database compound IDs to map to the 80 KEGG human reference pathways. The metabolites were ranked according to the *t*-test result. The Global Test for concerted change and the impact factor were calculated for these pathways. The impact factor used ‘betweenness’ centrality as the measure of impact, thus if altered, those molecules which act as hubs within a pathway contribute more strongly to the impact factor. Further functional analysis was conducted using the Ingenuity Pathway Analysis (IPA) core analysis of metabolites (QIAGEN, Redwood City, CA, USA; last accessed September 2014) is based on the Biocyc pathways and the proprietary Ingenuity knowledgebase. We used a p-value threshold of 0.05 and the set of all endogenous compounds as the reference group for the Fisher’s exact tests. The Human Metabolon Database number, the KEGG compound ID, or the PubChem ID numbers were used, with that order of preference, to map the metabolites to the IPA knowledgebase. Interactions from both experimentally validated and high confidence predictions were used. No restrictions were made on cell type or species.

## Results

### Metabolomic profile of platinum sensitive A2780 and resistant C200 cells lines

**Figure 1:**
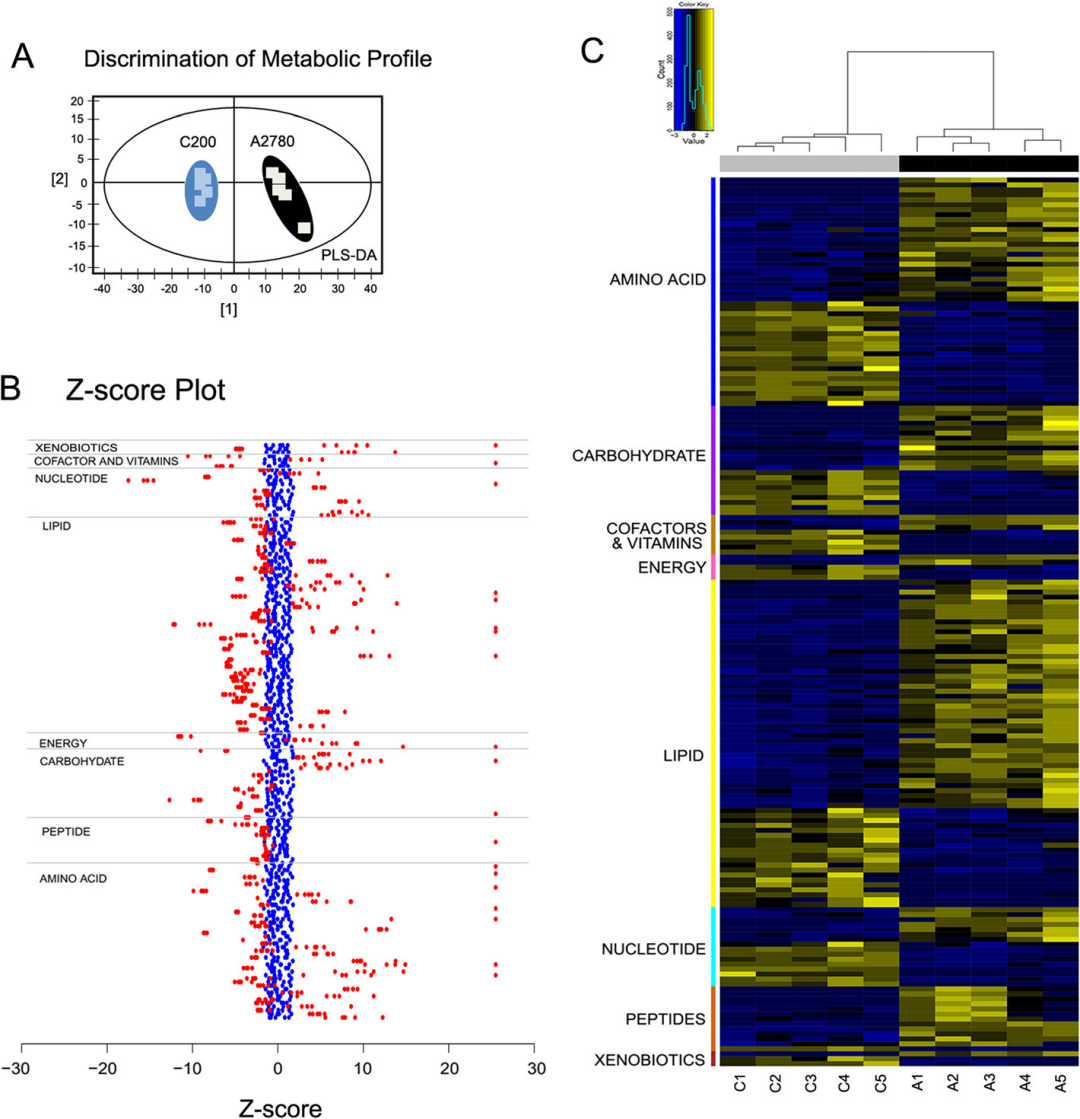
**Metabolomics profiling of platinum sensitive A2780 and resistant C200 cell lines. (A)** Partial least squares discriminant analysis (PLS-DA) score plot shows clear separation of metabolic profile of platinum sensitive A2780 and resistant C200. **(B)** Z-score plot of C200 metabolite intensities (RED, z-score range −18 to +174, truncated at 25) against A2780 metabolites (BLUE), taken as mean. Each dot represents 1 metabolite per observation. **(C)** Unsupervised hierarchical clustering of signature metabolites (N = 179) that separate A2780 vs C200 cells (*t*-test, p < 0.05). Yellow and blue indicate increased and decreased levels, respectively, with coloring for each metabolite relative to its mean observed intensity.

We performed a global metabolic analysis of the platinum sensitive ovarian cancer cell line A2780 and its isogeneic platinum resistant derivative cell line C200 [23]. For these cell lines 288 metabolites were identified. The missing value rate was only 10.6% per sample on average (range: 8.3%-13.2%). The PLS-DA combines features from principle component analysis and multiple regression and transforms a large number of potentially correlated variables into a smaller number of orthogonal variables (i.e. component 1, component 2) that discriminates between classes. We observed that a clear separation of A2780 and C200 cells can be achieved indicating that unique metabolite profiles are present for the sensitive and resistant cell lines (Figure 1A). The z-score plot showcases the extent of the metabolite alterations, where the intensities of the resistant C200 cells (red dots, z-score range −17 to +130, truncated at 25) are plotted relative to the distribution of the intensities in the sensitive A2780 cells (blue dots, Figure 1B). Each dot in the z-score plot represents 1 observation per single metabolite (rows), standardized against the mean and standard deviation of the A2780 cells and organized by major metabolic pathway. Formal testing, per-metabolite by two-sample t-tests, shows that there are 179 metabolites with differences in mean intensity at p < 0.05 (Additional file 1: Table S1). Of these, 70 were at higher concentrations and 109 were at lower concentration in the resistant C200, compared to the sensitive A2780 cells. The heatmap (Figure 1C) depicts the most altered metabolites (*t*-test p < 0.01). Here the metabolites (rows) are ordered by super-pathway and then by direction of change.

**Figure 2:**
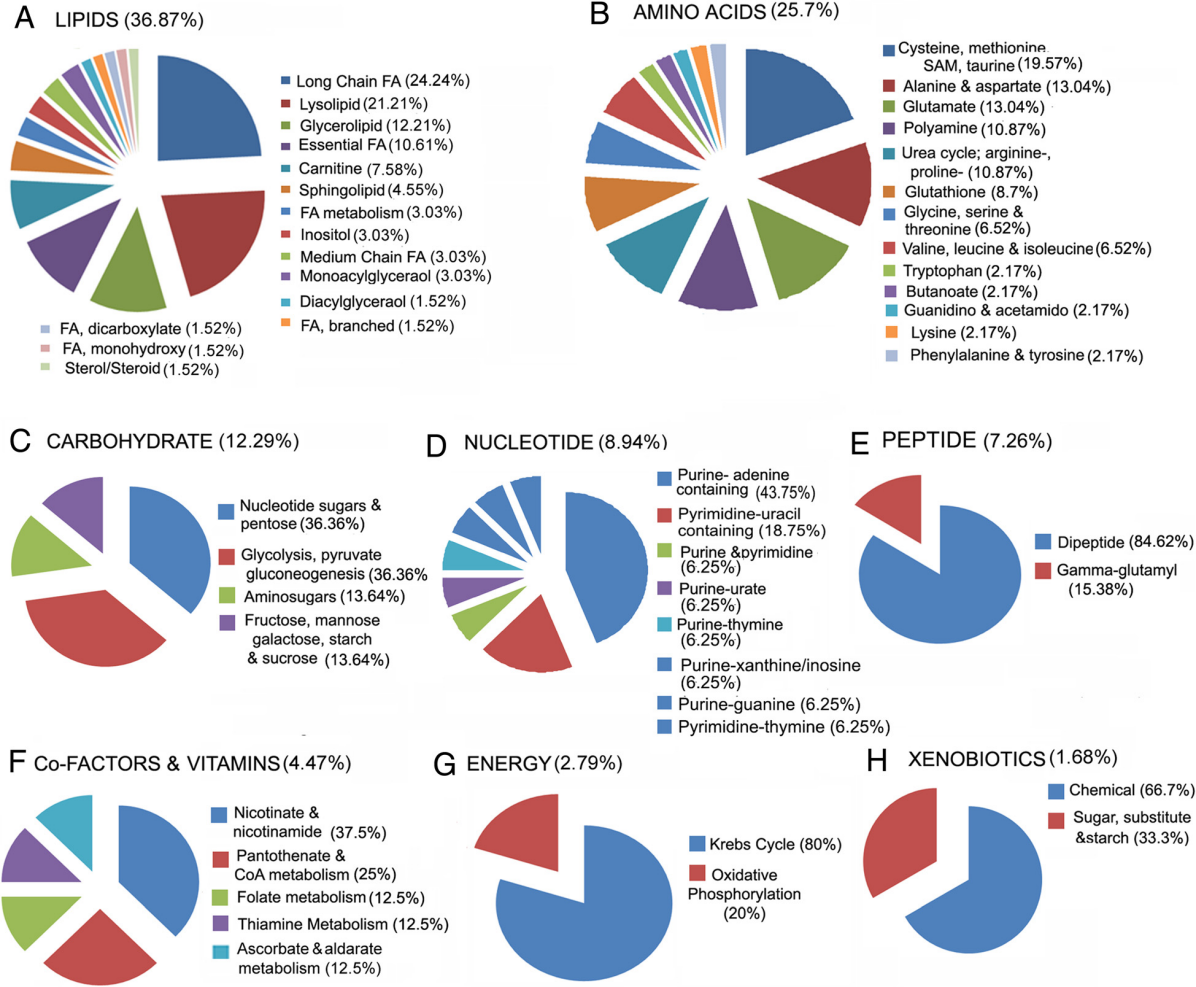
**Metabolic super-pathways and sub-pathways altered in platinum sensitive A2780 and resistant C200 cell lines.** To describe the altered metabolites observed in this study, each is classified into major classes of **(A)** Lipids, **(B)** Amino acids, **(C)** Carbohydrate, **(D)** Nucleotide, **(E)** Peptide, **(F)** Vitamins and co-factors, **(G)** Energy and **(H)** xenobiotics, then further into sub-pathways. CoA: Coenzyme A; FA: fatty acids; SAM: S-adenosylmethionine.

Collection of metabolites by super-pathway, revealed that most altered metabolites belonged to the lipid (36.87%) and amino acid (25.7%) pathways, followed by the carbohydrate (12.29%), nucleotide (8.94%), peptide (7.26%), cofactors and vitamins (4.47%), energy (2.79%), and xenobiotics (1.68%) (Figure 2). Metabolites were further classified into sub-pathways. Lipids showed the most changes in long chain fatty acids (24.24%) and lysolipids (21.21%) (Figure 2A). Metabolites of the cysteine, methionine, taurine (19.57%) and alanine-aspartate and glutamate (13.04% each) were most affected in the amino acids super-pathway (Figure 2B). Carbohydrate metabolites most altered belonged to the sub-pathways of nucleotide sugars and pentose (36.36%) and glycolysis-gluconeogenesis (36.36%) (Figure 2C). Purine metabolism–adenine containing (43.75%) sub-pathways were largely altered in the nucleotide super-pathways (Figure 2D). Dipeptides (84.62%) were most numerously changed in peptide pathway (Figure 2E). In energy metabolism, Krebs cycle metabolites (80%) were the most altered (Figure 2F). Amongst cofactors and vitamins, nicotinates (37.5%) were more altered (Figure 2G), while chemicals (66.67%) were more changed in the xenobiotic mediated metabolites (Figure 2H). Overall, the platinum sensitive A2780 and resistant C200 ovarian cancer cell lines showed distinct metabolite changes associated with various metabolic pathways.

### Metabolic pathway analysis of altered profiles

**Figure 3:**
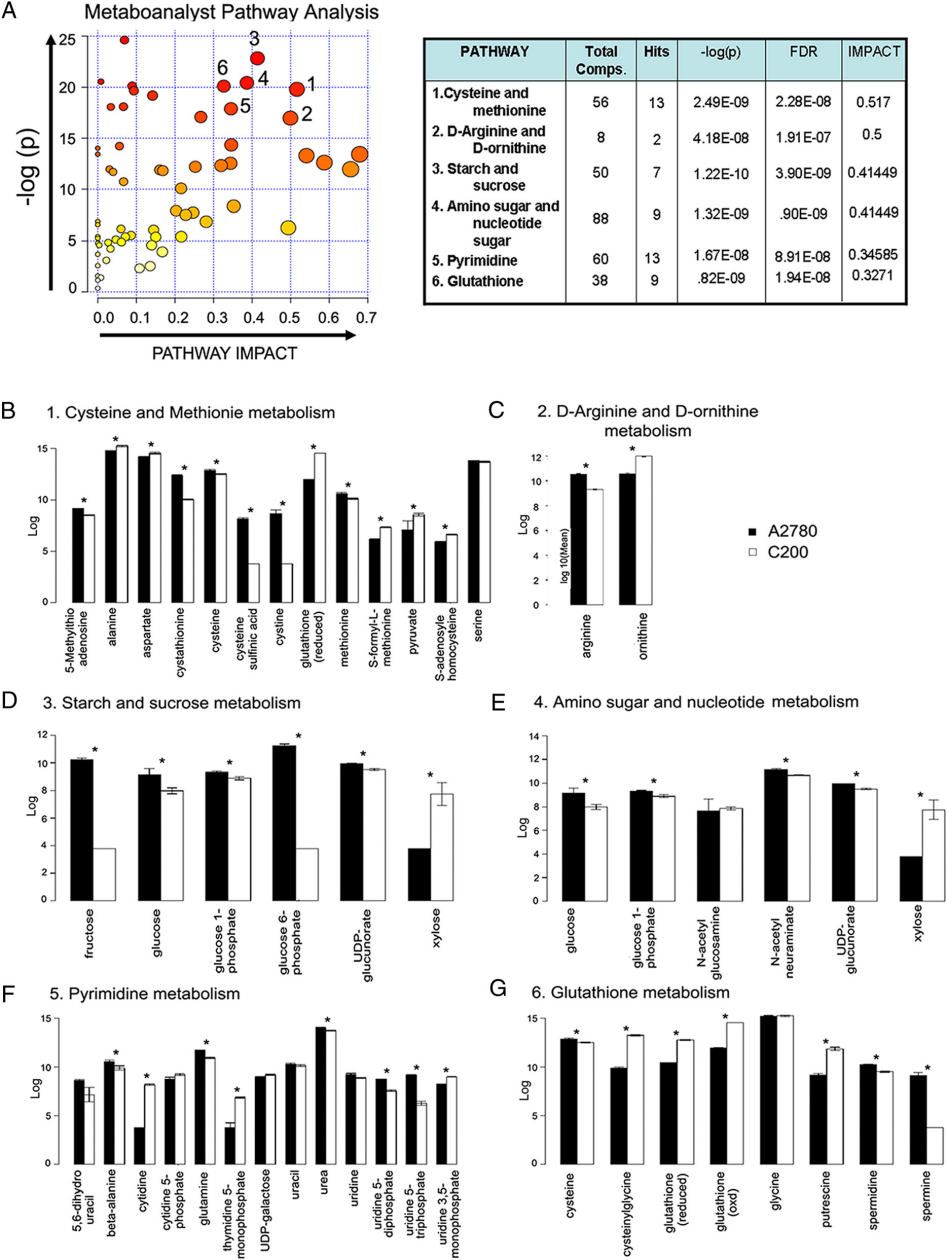
**MetaboAnalyst analysis. (A)** Human pathways of Kyoto Encyclopedia of Genes and Genomes (KEGG) (80) are simultaneously plotted to show the most significant pathways in terms of Global Test p-value (vertical axis, shades of red) and impact (horizontal axis, circle diameter). The top 6 pathways that arise with low p-values (−LN(P) > 15) and with “high” impact (impact > 0.3) are indicated in the table. Log mean of the individual metabolites altered within each metabolic pathway are represented as bar graphs. **(B)** Cysteine and methionine pathway; **(C)** D-arginine and ornithine metabolism; **(D)** starch and sucrose metabolism; **(E)** amino sugar and nucleotide sugar metabolism; **(F)** pyrimidine metabolism; and **(G)** glutathione metabolism. *p < 0.05. Abbreviations: Comps: compounds; FDR: false discovery rate.

To understand the functional role of the profile alterations, the KEGG metabolic library [24] was analyzed using MetaboAnalyst [25] (Figure 3). Of the 288 measured metabolites, 73% were able to be mapped to the KEGG pathways. Both the correlated change of metabolite intensities within pathway between condition (Global Test) [26], and the impact of the changed metabolites on the function of the pathway through alterations in critical junction points of the pathway (relative betweenness centrality) were assessed. Results of each of the 80 human pathways of KEGG were simultaneously plotted (Figure 3A) to show the most significant pathways in terms of Global Test p-value (vertical axis, shades of red) and impact (horizontal axis, circle diameter), Additional file 2: Table S2 [24]. The top 6 pathways that emerged with low p-values (−LN(P) > 15) and with “high” impact (impact > 0.3) are indicated in Figure 3A: 1) cysteine and methionine; 2) D-arginine and ornithine; 3) starch and sucrose; 4) amino sugar and nucleotide; 5) pyrimidine; and 6) glutathione (GSH) pathways. The log mean concentrations of the metabolites considered under each pathway are depicted in Figure 3B-F. The significantly altered metabolites categorized under cysteine and methionine metabolism included: 5-methylthioadenosine, cystathione, cysteine, cysteine sulfunic acid, cysteine and methionine that were present in reduced levels in the resistant C200 cells compared to A2780 cells; while alanine, aspartate, reduced GSH, S-formyl-L-methionine and pyruvate were increased in C200 cells (Figure 3B). Arginine was reduced and ornithine levels were at increased levels in the resistant cells (Figure 3C). Most of the metabolites grouped under starch and sugar metabolism and amino sugar and nucleotide metabolism were same as earlier pathway categories (fructose, glucose, glucose 1-phosphate, glucose 6-phosphate, N-acetyl neuraminate and UDP-glucunorate), and were decreased in the resistant C200 cells, except xylose and N-acetyl glucosamine, which were increased (Figure 3D and E). Pyrimidine metabolites 5,6-dihydro uracil, beta-alanine, glutamine, urea, uridine 5-diphosphate and uridine 5-triphosphate were significantly lower in resistant C200 cells while cytidine, thymidine 5-monophosphate and uridine 3,5-monophosphate were significantly higher compared to sensitive A2780 cells (Figure 3F). Metabolites of the GSH metabolism, including cysteine, cysteinylglycine, reduced GSH and oxidized GSH, which are downstream products of the cysteine metabolism, were at higher concentrations in the C200 cells (Figure 3G). Metabolite products from the polyamine biosynthesis included putrescine, which was increased, and spermidine and spermine, which were decreased in the resistant C200 compared to the sensitive A2780 cells (Figure 3G). Thus, the most altered metabolic pathways shared many common metabolites, suggesting a link between the important altered metabolic pathways.

### Ingenuity pathway analysis (IPA)

**Figure 4:**
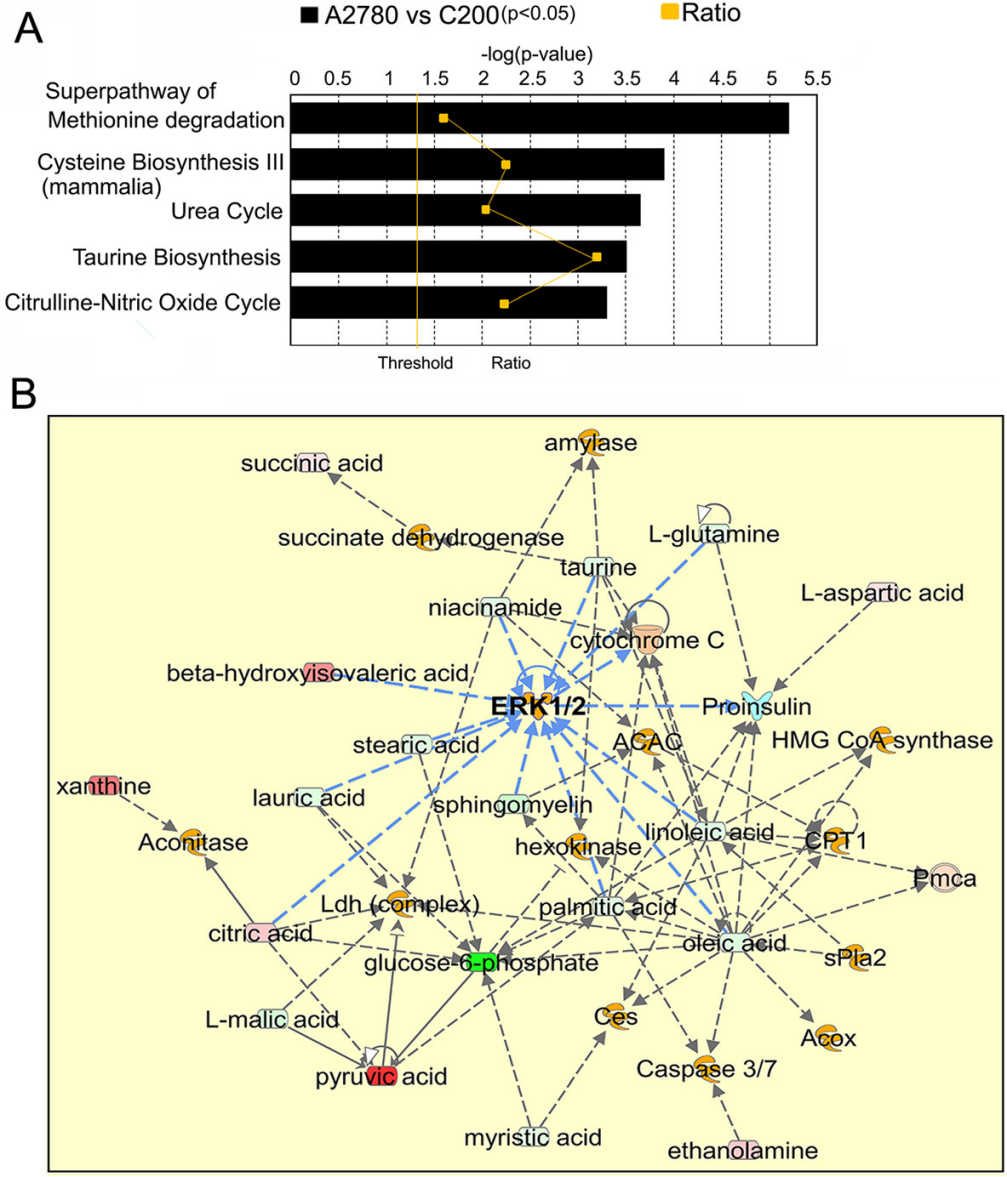
**Ingenuity Pathway Analysis (IPA) analysis. (A)** Top 5 canonical pathways enriched by observed metabolite alterations. **(B)** The top network includes aspects of carbohydrate metabolism, energy production, and small molecule biochemistry. Networks, constructed from the IPA knowledgebase by connecting the altered molecules, are not limited by canonical pathway boundaries. Abbreviations: ERK 1/2: extracellular-signal-related kinases 1 and 2; CPT1:.carnitine palmitoyltransferase 1; ACAC: acetyl CoA carboxylase; sPLA2: soluble phospholipase A2; PmCa: plasma membrane calcium ATPase; HMGCoA: 3-hydroxy-3-methyl-glutaryl-CoA; Acox: acyl CoA; Ces: cholesterol esters.

To gain biologically related molecule networks, 217 of 288 molecules were mapped into the IPA knowledgebase. The top 5 canonical pathways observed were 1) super-pathway of methionine degradation; 2) cysteine biosynthesis III; 3) urea cycle; 4) taurine biosynthesis; and 5) citrulline-nitric oxide cycle (Figure 4A; Additional file 3: Table S3). The methionine degradation and cysteine biosynthesis pathway metabolite alterations align with the cysteine and methionine metabolism pathway that had the lowest p-value and the most impact value in the MetaboAnalyst analysis (Figure 3), further supporting that it could be the most significant metabolic pathway in our system. The top 5 altered bio-functions in terms of molecular and cellular functions were cell signaling (p-value range: < 0.0001 to 0.0335); molecular transport (p-value range: < 0.0001 to 0.0409); vitamin and mineral metabolism (p-value range: < 0.0001 to 0.0386); lipid metabolism (p-value range: < 0.0001 to 0.0386), and small molecule biochemistry (p-value range: < 0.0001 to 0.0386) (Additional file 3: Table S3).

IPA network analysis, using the proprietary Ingenuity knowledgebase, constructs networks of altered molecules which are not limited by canonical pathway boundaries. Eleven networks were constructed as reported in Additional file 3: Table S3. The top network uses 19 molecules and focuses on carbohydrate metabolism, energy production, and small molecule biochemistry (Figure 4B). Containing metabolites like glucose-6-phosphate (down in C200), pyruvate (high in C200) and citric acid (high in C200) from energy metabolism pathway and various lipids like oleic acid, malic acid, palmitate, linoleic acid, etc. (all low in C200); it has hubs at the signaling molecule extracellular-signal-related kinases1/2 (ERK) (Figure 4B). ERKs participate in the Ras-Raf-MEK-ERK signaling pathway and is reported to be up-regulated in almost all cancers [27]. Network 2 uses 20 molecules and focuses on drug metabolism, molecular transport, and small molecule biochemistry. Most of the metabolites in this network are derived from the methionine-cysteine metabolism and related to amino acids that centralize on GSH (up in C200) and Akt as the signaling molecule (Additional file 4: Figure S1), that is a constituent of one of the most dysfunctional signal transduction pathways described in various cancers [28]. Network 3 included 16 metabolites of pyrimidine metabolism to generate a linkage of nucleic acid metabolism and small molecule biochemistry (Additional file 5: Figure S2, Additional file 3: Table S3). The signaling hubs were calcium ions and epidermal growth factor receptor. Network 4 uses 15 molecules and focuses on free radical scavenging, lipid metabolism and small molecule biochemistry. Metabolites in this network are from methionine metabolism, phospholipids and nucleic acid metabolism, superoxide and GSH (Additional file 6: Figure S3). Network 5 with 12 metabolites was convened as carbohydrate metabolism, lipid metabolism and molecular transport. All metabolites except sn-glycerol3-phosphate were lower in C200 cells, including the central molecule, D-glucose. The lipids stearic acid and cholesterol were also lower in the C200 cells. The network revealed connections leading to pro-oncogenic signaling molecules like mammalian target of rapamycin complex 1 (mTORC1), c-Jun N-terminal kinases, VEGF and Peroxisome proliferator activator receptor (Additional file 7: Figure S4). Overall, the networks analysis suggests that altered metabolites of energy metabolism, methionine metabolism and lipids are linked with tumor promoting signaling molecules.

## Discussion

**Figure 5:**
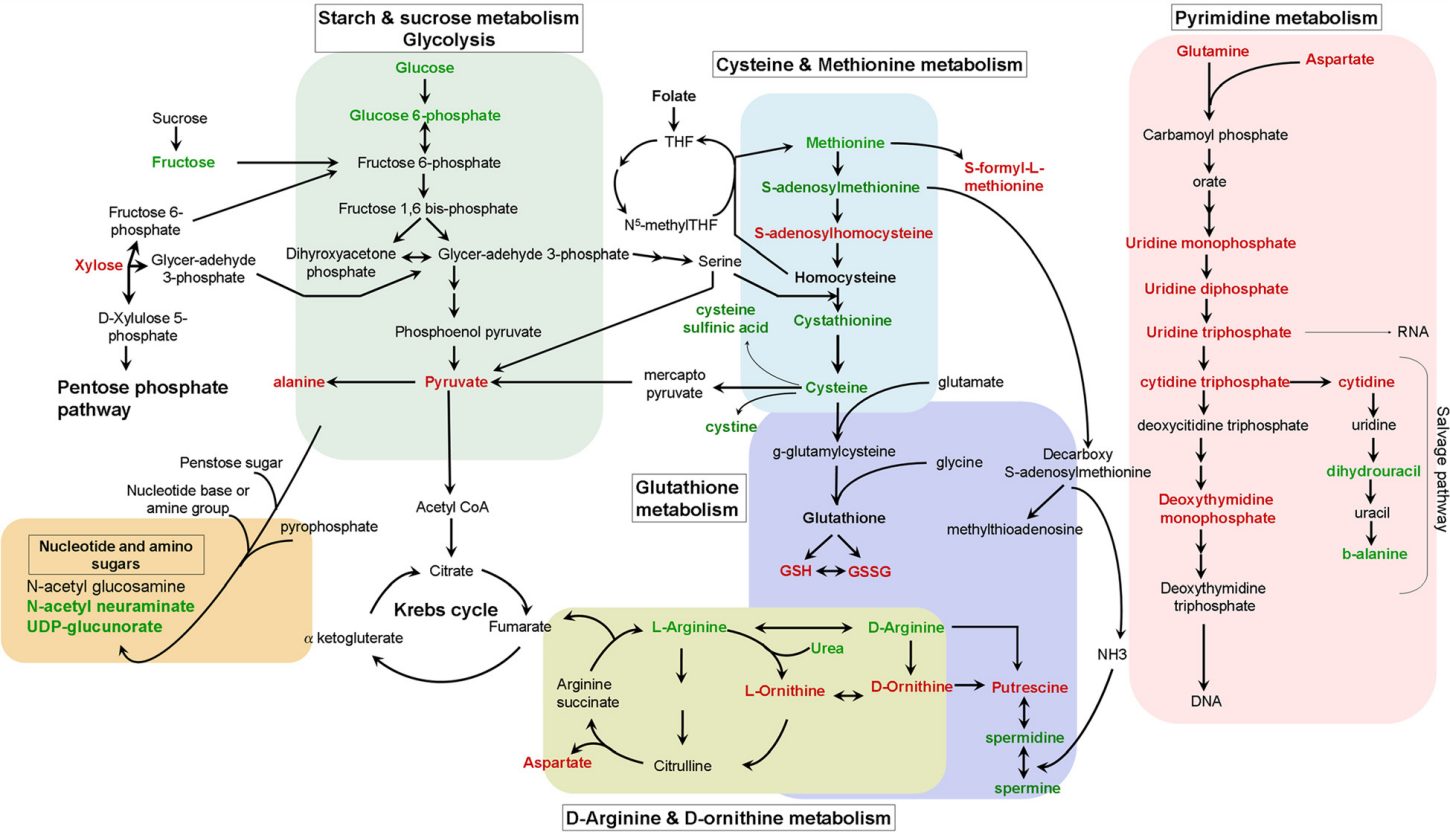
**Interconnected altered metabolic pathways.** Altered metabolites from the top 6 MetaboAnalyst analysis and the top 3 Ingenuity Pathway Analysis are charted within their own metabolic pathway along with their cross-participation in other metabolic pathways. RED text metabolites represent elevated mean intensity in C200 cells compared to A2780 cells, whereas GREEN text represent reduced intensity. Abbreviations: CoA: Coenzyme A; THF: tetrahydrofolate; GSH: reduced glutathione; GSSH: oxidized glutathione; UDP: uridine diphosphate.

Our metabolomics analysis demonstrated that distinct metabolic profiles can be detected for platinum sensitive A2780 and resistant C200 ovarian cancer cells (Figure 1). The altered metabolites encompassed all major molecular categories and metabolic pathways (Figure 2) and appeared to be inter-connected. Arranging the significantly altered metabolites from the KEGG metabolic pathways and the top canonical pathways from IPA into a single map revealed a high level of connectivity and interdependence among the pathways (Figure 5). For example, the glycolysis-to-citrate cycle not only generates adenosine triphosphate, which is required for all biosynthetic pathways to occur, but also synthesizes essential metabolites such as glyceraldehyde 3-phosphate, a precursor for the generation of amino acid serine. Serine then enters the methionine pathway to produce cysteine, leading to GSH production. GSH, apart from protecting cells from oxidative stress, also maintains the NADPH/NADP+ ratio, which is required by the folate cycle and nucleotide biosynthesis, among others [29]. Cysteine can be converted back to pyruvate by transamination [29]. The glycolysis-to–pentose phosphate pathway metabolites are also the sources for precursors of nucleotides and modifiers of lipids, purine and protein modifications. Thus the metabolism of a cell runs its own ‘cycle of life’ in unison that sustains the cell function and growth. Most of the altered metabolites participate in more than 1 pathway in significant ways and the change in that 1 metabolite could have a resonating effect for other pathways.

**Figure 6:**
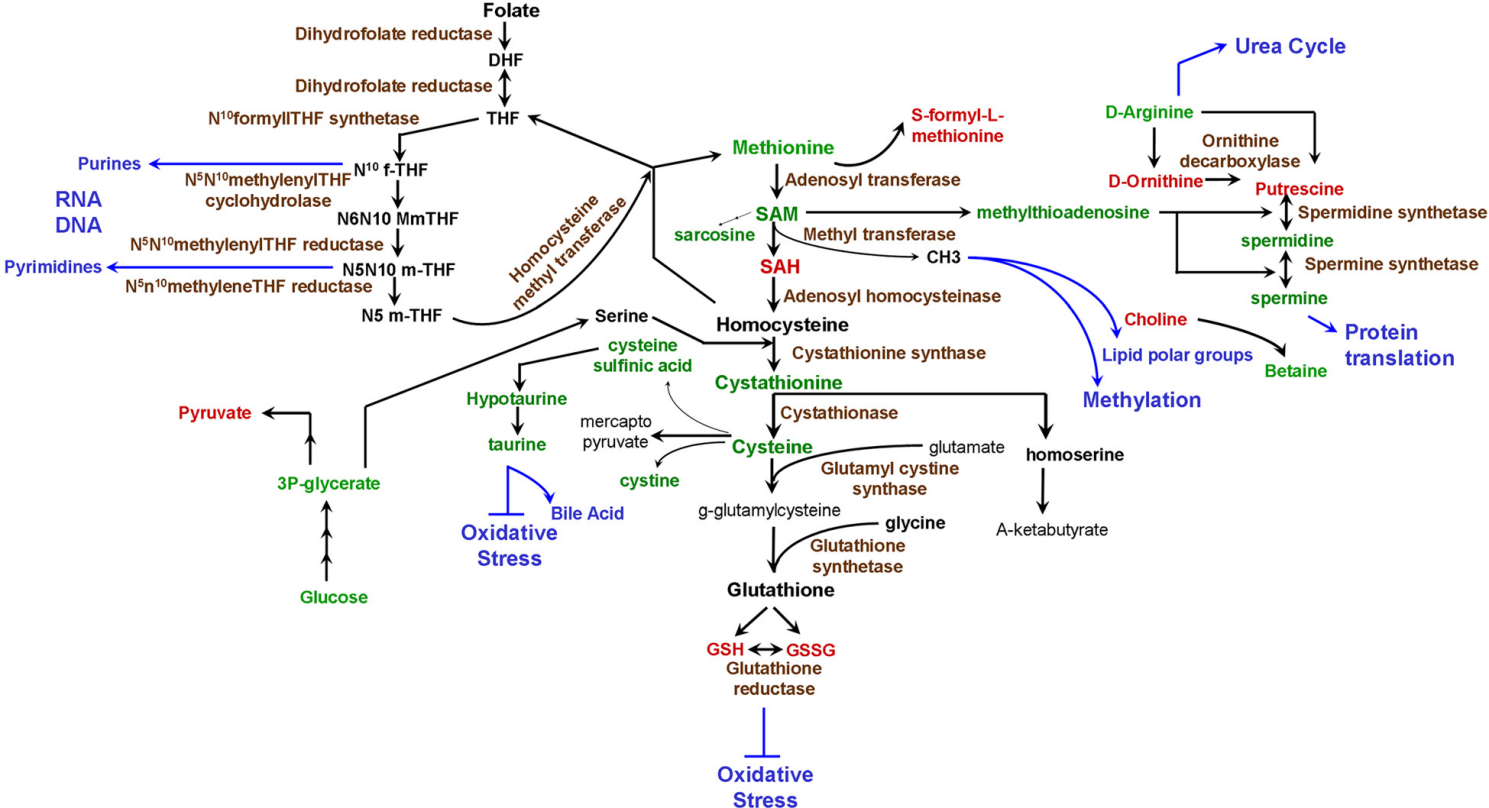
**Methionine metabolism pathway.** A bio-chart of the methionine metabolism pathway and the related pathways of folate, glutathione and polyamine biosynthesis. RED text metabolites represent elevated mean intensity in C200 cells compared to A2780 cells, whereas GREEN text represent reduced intensity. The catalyzing enzymes are texted BROWN. BLUE arrows and text convey the requirement of that pathway metabolite towards the indicated macromolecule synthesis. Abbreviations: DHF: dihydrofolate; THF: tetrahydrofolate; f-THF: formate-THF; m-THF: methylene-THF; SAM: S-adenosylmethionine; SAH: S-adenosylhomocysteine; CH3: methyl group; GSH: glutathione, GSSG: oxidized glutathione.

Collectively the largely significant altered metabolites belonged to the methionine-cysteine metabolism and one of its downstream metabolic tributaries of the GSH pathway (Figure 6). The methionine pathway along with folate and transulfuration pathways constitutes the one-carbon metabolism [29,30]. The one-carbon metabolism is known as a metabolic integrator that assimilates products from the glucose pathway and amino acids to fulfill integral and essential cellular biosynthesis for biological functions, such as cellular turnover and proliferation. The one-carbon metabolism pathway has also been investigated as a major contributor to the process of oncogenesis [30]. Studies have shown that methionine levels are directly involved in promoting proliferation of cancer cells [31] and in protecting the cancer cells against chemotherapeutic drugs like Fluorouracil [32]. Methionine deprivation has been shown to inhibit tumor cell growth in various cancers both in *in vitro* and *in vivo* models along with chemo-sensitizing cancer cells [33-36]. We observed decreased levels of methionine in resistant C200 cells, which could indicate its increased utilization (Figure 3B), and could suggest the presence of ‘methionine dependency’ in the resistant cells. Methionine-dependency is defined as inability or reduced ability of cancer cells to proliferate even when methionine is replaced by its precursor homocysteine, this phenotype has been demonstrated in several cancers like prostate and lymphoma [37,38]. Since methionine is an integral participant of various metabolic pathways, the exact mechanism underlying methionine-dependence of various cancer cells has been difficult to elucidate [37]. Homocysteine is an important metabolite of the methionine pathway that is coupled with the folate cycle and on gaining a methyl group from methyl THF converts back to methionine [29,39]. Methionine can also be produced by the salvage pathway via the crucial enzyme methylthioadenosine phosphorylase (MTAP), which has been shown to be deleted in various cancers [40]. Methylthioadenosine, the substrate for MTAP, was observed to be present in lower quantities in C200 cells compared to A2780 (Figure 3B), which could again suggest use and diversion of methionine to other pathway end-product metabolites like GSH or putrescine (discussed below), rather than the normal metabolic cycle. Homocysteine through transsulfuration reactions also gives rise to cysteine. One of the most important roles for cysteine is to act as the limiting factor for synthesis of the antioxidant GSH [39,41,42]. GSH is the main endogenous antioxidant and protects the cells from metabolic stresses by nonenzymatically reducing substances like peroxides and free radicals and maintaining an intracellular reducing environment. It also activates GSH s-transferase and detoxifies xenobiotics and other cell damaging compounds [42]. GSH has been established as a protective mechanism against the increased oxidative stress encountered by the cancer cells [42,43], which has been implicated as a contributing factor to chemoresistance development in cancer cells [41]. GSH is present in 2 forms: the reduced and the oxidized GSH disulfide forms. C200 cells displayed higher levels of both forms of GSH with the oxidized form being higher than the reduced form (Figure 3G). This could indicate a higher level of oxidative stress in the C200 cells and a very active GSH mediated antioxidant system that is offering added protection to the cells. Thus is our system, the lowered methionine levels could also indicate its eventual utilization to maintain the high levels of GSH observed in the C200, as a means to encounter the assault of chemotherapeutic drugs.

A central metabolite S-adenosylmethionine (SAM) from the methionine pathway, acts as a donor for methylation reactions involving methylation of histones, DNA, RNA and all general protein methylations [39,44], and also participates in biosynthesis of phosphatidylcholine, the major component of cell membranes, by forming the polar head group with choline [30]. Methylation processes have been widely implicated in cancer progression, including ovarian [45,46]. Aberrant methylation has also been proposed as a contributing factor for acquisition of chemoresistance, especially in resistance against DNA-damaging platinum drugs [47,48]. Recently, a cisplatin-resistant cell line derived from the sensitive A2780 ovarian cancer cell lines were shown to preferentially select for DNA-hypermethylation and the obtained methylated gene signature was found to partially hold validation in a small subset of patient relapsed ovarian tumors [49]. Thus it is possible that the chemoresistance in C200 ovarian cancer cells could be a result of increased methylation of selective genes, which could be reflected in its altered metabolism. SAM also provides methyl groups for biosynthesis of polyamines, a vital class of products involved in cell proliferation, and have been shown to be increased during malignancy [50]. Polyamines include putrescine and its derivatives spermidine and spermine, which are synthesized from ornithine and shown to be required for proliferation [50-52]. On one hand, inhibition of polyamine synthesis has been shown to inhibit cancer cells, but on the other hand recent studies have reported decreased levels of spermine as a metabolic biomarker for cancer cells [50,53]. We observed an increased level of putrescine and decreased levels of spermine and spermidine (Figure 3G). This could either indicate a block after putrescine formation or increased utilization of these metabolites. Ornithine decarboxylase (ODC) is the first enzyme required for polyamine synthesis and is regulated by Myc oncogene [50,54]. Induction of spermidine/spermine N1-acetyltransferase 1(SAT1), the key enzyme regulating catabolism of polyamines has been reported to result in decreased spermine and spermidine and increased putrescine levels [55], similar to our observation in C200 cells (Figure 3G). ODC and SAT1 have been shown to be overexpressed in neoplastic prostate tissue [56]. Platinum drugs have also been shown to regulate polyamine metabolism enzymes including SAT1 [18]. Thus acquisition of platinum resistance in C200 cells could be related to perturbations in the enzymatic makeup of polyamine metabolism.

Our observations are similar to other studies where the altered methionine pathway has been advocated to play a role in ovarian and other cancers and its metabolites presented as biomarkers. Alteration of methionine pathway was suggested early in ovarian cancer [57], where accumulated homocysteine in ascites was an indicator of malignancy. A recent study has shown cisplatin treatment of embryonic mouse cells to induce significant changes in the methionine degradation pathway, including GSH and polyamine metabolism, along with other methionine associated pathways [18]. Sarcosine (n-methylglycine), a product of the methionine degradation pathway was reported as a biomarker in urine of metastatic prostate cancer patients [19]. Comparison of metabolites from early recurrence (within 2 years of surgery) and recurrence free (more than 5 years) prostate cancer patients found elevated products of methionine catabolism in serum, which included sarcosine, cysteine, cystathionine and homocysteine [58]. Targeting of the methionine pathway is being actively investigated in cancer therapeutics. Targeting the epigenetic status by inhibiting methylation of various genes in tumors is one of the earliest and most pursued chemotherapeutic approaches [59-61]. SAM-mediated methylation and polyamine synthesis is being actively investigated in preclinical studies of various cancers [53,62]. Inhibition of GSH activity is also a major area under consideration for specific targeting of chemoresistance [42]. Thus the methionine pathway metabolites appear to have the potential to act as biomarkers specifically for metastatic or recurrent tumors. Together, with the observation that most recurrent tumors are also chemoresistant and our findings, surfacing of the methionine pathway as an indicator of chemoresistance in ovarian cancer is significant. Thus, an in-depth analysis of the methionine metabolism encompassing and integrating the levels of input, intermediate and outcome metabolites flux, along with the expression and activation status of the enzymes catalyzing these reactions will provide a complete picture in understanding the functional significance of this pathway in platinum chemoresistance of ovarian cancer.

**Figure 7:**
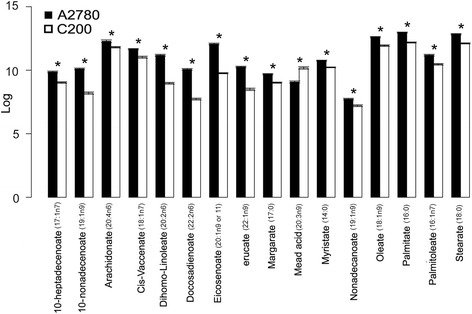
**Long chain fatty acid levels.** Log10 mean of the significantly (p < 0.05) altered individual long chain fatty acids and their metabolites are represented as bar graphs.

While lipids were the most altered class of compounds (36.87%) observed between the resistant and sensitive cell lines, they were not well represented in the KEGG pathways. However, 78% of the lipids had at least 1 identifier recognizable by IPA where lipid metabolism was the fourth altered biofunction. Additionally, all of the top 5 networks had substantial participation of lipid metabolites, related enzymes and downstream effectors. Approximately 70% of the lipids were decreased in C200 cells, which could be suggestive of a decreased turnover or a decrease in the metabolism of membrane lipids. Decreased circulating phospholipids (plasmenylphosphoethanolamine and lysophosphadylcholine) and increased levels of lysophosphatidic acid (LPA) have been proposed as biomarkers for ovarian cancer [63,64]. LPA has been shown to be a potent mitogen for ovarian cancer cells and are found in increased amount within ascites [63]. Plasmenylphosphoethanolamine can act as an antioxidant and its absence may be a consequence of a higher oxidative environment associated with chemoresistant cells. Lysophosphadylcholine is a main membrane component and could be utilized as a building block for increased proliferation of cancer cells [63]. The largest group affected was the long chain fatty acids, which included palmitate, oleate, stearate, etc. (Figure 7). Alternatively, since fatty acids are the major building blocks for the synthesis of triacylglycerides, which are mainly used for energy storage, this could also indicate an increased breakdown of the fatty acids by the mitochondria for energy production. The IPA network analysis projected energy metabolism and lipid metabolism metabolites as contributors in 3 of the 5 top networks (Figures 4B and Additional file 7: Figure S4; Additional file 3: Table S3). A closer look at the involved molecules supports an altered energy metabolism between the resistant C200 and sensitive A2780 cells. The C200 cells have higher levels of metabolites from the tricarboxylic acidcycle (pyruvate, citric acid and succinic acid) and lower fatty acids (palmitic, oleic and linoleic acid) and links to the enzymes from the respective pathways (tricarboxylic acid cycle: aconitase, succinate dehydrogenase, lactate dehydrogenase; fatty acid oxidation: Carnitine palmitoyltransferase-1, Acetyl CoA Carboxylase) (Figure 6). Therefore the lower levels of overall lipids may be indicative of either decreased biosynthesis or increased utilization of lipids. This could suggest a further alteration in the energy metabolism of the chemoresistant cells, along with the already altered metabolism established in cancer cells [65].

## Conclusions

The altered metabolism of cancer cells has been well established. Our data shows that platinum sensitive and resistant ovarian cancer cells can be distinguished based on their metabolite profiles. This suggests that chemoresistance is associated with its own set of metabolic changes that can be exploited for biomarker and targeted therapeutic approaches. Based on our data, we propose the altered methionine pathway metabolites as the potential biomarkers for platinum resistance in ovarian cancer cells. Most of these metabolites are measurable in body fluids like urine or serum, which would make their translation into clinical practice easier, once they have been extensively validated in subsets of ovarian cancer patients. Although our study is limited by using an isolated *in vitro* cell line system, we observed alterations in metabolites similar to those reported to be associated with malignancy, specifically metastatic and recurrent tumors. While our data await further validation, a comprehensive analysis of the altered pathways may not only provide biomarkers of chemoresistance but can also provide clues of the biology underlying platinum resistance in ovarian cancer cells and offer plausible therapeutic targets to specifically target chemoresistance

## Additional files

Additional file 1: Table S1. Spreadsheet file of all raw metabolite data. Additional file 2: Table S2. Spreadsheet file of MetaboAnalyst analysis results. Additional file 3: Table S3. Spreadsheet file of Ingenuity Pathway Analysis core analysis results. Additional file 4: Figure S1. Ingenuity Pathway Analysis (IPA) network 2. The second network contains components of drug metabolism, molecular transport, and small molecule biochemistry. Constructed from the IPA knowledgebase using altered molecules, these networks are not limited by canonical pathway boundaries. Abbreviations: ALT : alanine amino transferase; CYP: cytochrome P450; GABA: gamma –aminobutyric acid; HDL: high- density lipoprotein; LDL: low density lipoprotein; Sod: superoxide dismutase; Ggt: gamma-glutamyl transferase; Na: Sodium. Additional file 5: Figure S2. Ingenuity Pathway Analysis (IPA) network 3. The third network contains components of nucleic acid metabolism and small molecule biochemistry. Constructed from the IPA knowledgebase using altered molecules, these networks are not limited by canonical pathway boundaries. Abbreviations: ITLN1: interlectin 1; CHRNA1: cholinergic receptor, nicotin alpha 1; Ca: calcium; PKC: protein kinase C; DuoX1: dual oxidase 1; EGFR: epidermal growth factor receptor 1; IFNG: interferon gamma; THOP1: human endopeptidase 1; APOBEC: DNA cytosine deaminase; AICDA: activation induced cytidine deaminase; CDA: cytidine deaminase. Additional file 6: Figure S3. Ingenuity Pathway Analysis (IPA) network 4. The fourth network contains components of free radical scavenging, lipid metabolism and small molecule biochemistry. Constructed from the IPA knowledgebase using altered molecules, these networks are not limited by canonical pathway boundaries. Abbreviations: Cu: copper; UDP: uridine diphosphate; MTUS1: mitochondrial tumor suppressor 1; NDUFS1: NADH-ubiquinone oxidoreductase iron-sulfur protein 1; CDKN1A: cyclin dependent kinase inhibitor 1A; CYP2C8: cytochrome 2C8; SLC22A6: solute carrier family 22 member 6. Additional file 7: Figure S4. Ingenuity Pathway Analysis (IPA) network 5. The fifth network contains components of carbohydrate metabolism, lipid metabolism and molecular transport. Constructed from the IPA knowledgebase using altered molecules, these networks are not limited by canonical pathway boundaries. Abbreviations: DPP4: dipeptidyl peptidase 4; VEGF: vascular endothelial growth factor; MTORC1: mammalian target of rapamycin complex1; JNK: c-jun N-terminal kinase; PPARA: peroxisome proliferator-activated receptor alpha; IDH1: isocitrate dehydrogenase 1; MOGAT2:acyl CoA: monoacylglycerol acyltransferase; TALDO1: transaldolase1; FBP2: phosphofruckokinase 2; CCK: cholecystokinin; PFKFB1: 6-phosphofructo-2-kinase/fructose-2,6-biphosphatase 4; HSD11B1: 11-beta-hydroxysteroid dehydrogenase type 1; TKTL1: transketolase 1; ATP5G2: ATP synthase; PL1N2: perilipin 2; CA5A: carbonic anhydrase VA.

## Abbreviations

ACAC: Acetyl CoA carboxylase
AICDA: Activation induced cytidine deaminase
APOBEC: DNA cytosine deaminase
Acox: Acyl CoA
ALT: Alanine amino transferase
ATP: Adenosine triphosphate
ATP5G2: ATP synthase
Ca: Calcium
CA5A: Carbonic anhydrase VA
CCK: Cholecystokinin
CDA: Cytidine deaminase
CDKN1A: Cyclin dependent kinase inhibitor 1A
Ces: Cholesterol esters
CH3: Methyl group
CoA: Coenzyme A
CHRNA1: Cholinergic receptor, nicotin alpha 1
CPT1: Carnitine palmitoyltransferase 1
CYP: Cytochrome P450
Cu: Copper
DHF: Dihydrofolate
DPP4: Dipeptidyl peptidase 4
DuoX1: Dual oxidase 1
EGFR: Epidermal growth factor receptor 1
ERK: Extracellular-signal-related kinases
FA: Fatty acids
FBP2: Phosphofruckokinase 2
f-THF: Formate tetrahydrofolate
GABA: Gamma –aminobutyric acid
Ggt: Gamma-glutamyl transferase
GSH: Glutathione
GSSG: Oxidized glutathione
HDL: High- density lipoprotein
HMGCoA: 3-hydroxy-3-methyl-glutaryl-CoA
HSD11B1: 11-beta-hydroxysteroid dehydrogenase type 1
IDH1: Isocitrate dehydrogenase 1
IFNG: Interferon gamma
IPA: Ingenuity Pathway Analysis
ITLN1: Interlectin 1
JNK: c-jun N-terminal kinase
KEGG: Kyoto Encyclopedia of Genes and Genomes
LDL: Low density lipoprotein
MOGAT2: Acyl CoA: monoacylglycerol acyltransferase
m-THF: Methylene tetrahydrofolate
MTORC1: Mammalian target of rapamycin complex1
MTUS1: Mitochondrial tumor suppressor 1
Na: Sodium
NADH: Nicotinamide adenine dinucleotide, hydrogen ion
NDUFS1: NADH-ubiquinone oxidoreductase iron-sulfur protein 1
PFKFB1: 6-phosphofructo-2-kinase/fructose-2,6-biphosphatase 4
PKC: Protein kinase C
PL1N2: Perilipin 2
PLS-DA: Partial least squares discriminant analysis
PmCa: Plasma membrane calcium ATPase
PPARA: peroxisome proliferator-activated receptor alpha
SAH: S-adenosylhomocysteine
SAM: S-adenosylmethionine
SLC22A6: Solute carrier family 22 member 6
sPLA: Soluble phospholipase A2
Sod: Superoxide dismutase
TALDO1: Transaldolase1
THOP1: Human endopeptidase
1THF: Tetrahydrofolate
TKTL1: Transketolase 1
UDP: Uridine diphosphate
VEGF: Vascular endothelial growth factor
