# Supplementary figures and images for: A metabolomic approach to identifying platinum resistance in ovarian cancer
Source: J Ovarian Res. 2015 Mar 26;8:13. doi: 10.1186/s13048-015-0140-8 (PMC4396147; doi:10.1186/s13048-015-0140-8)

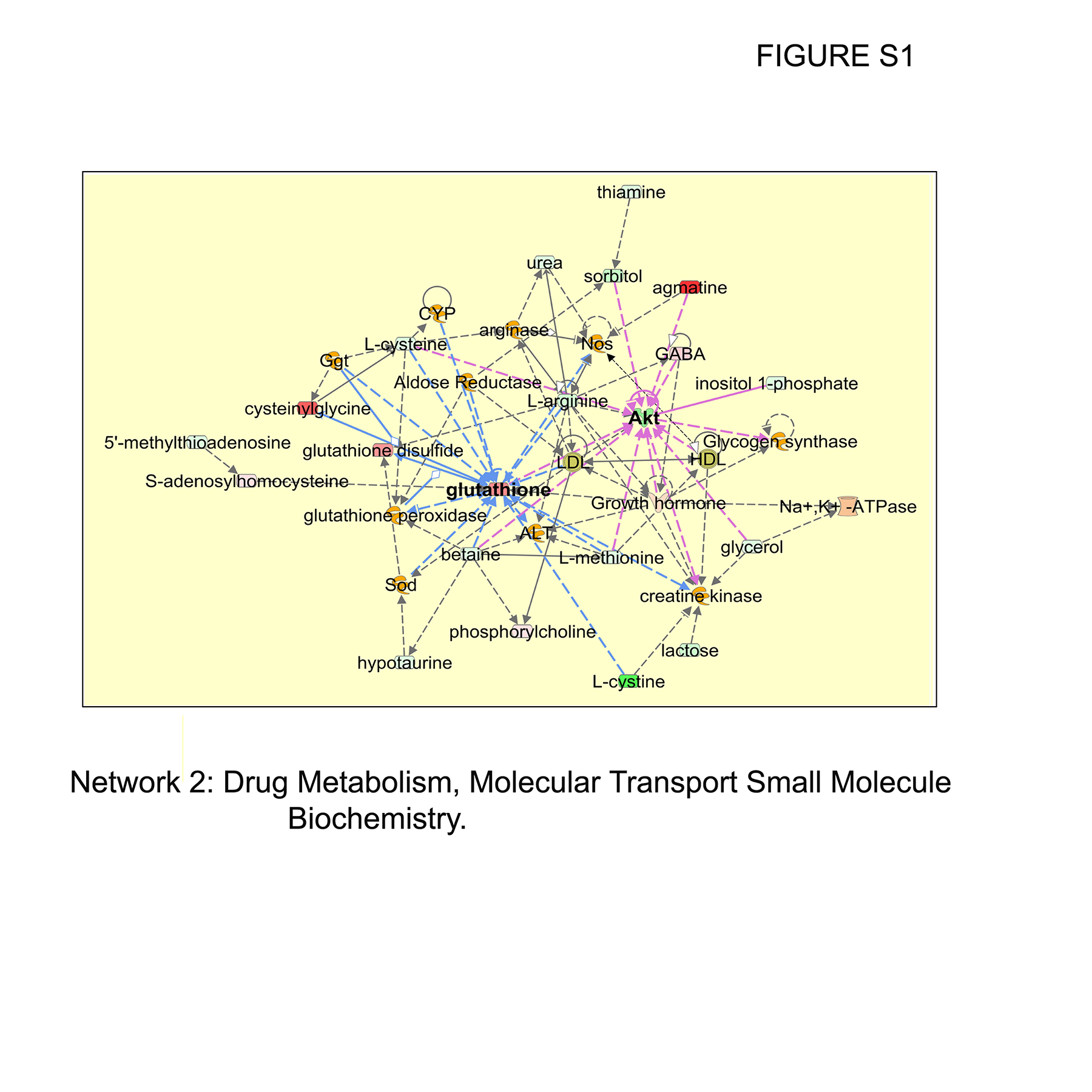

Supplement: Additional file 4: Figure S1. — Ingenuity Pathway Analysis (IPA) network 2. The second network contains components of drug metabolism, molecular transport, and small molecule biochemistry. Constructed from the IPA knowledgebase using altered molecules, these networks are not limited by canonical pathway boundaries. Abbreviations: ALT : alanine amino transferase; CYP: cytochrome P450; GABA: gamma –aminobutyric acid; HDL: high- density lipoprotein; LDL: low density lipoprotein; Sod: superoxide dismutase; Ggt: gamma-glutamyl transferase; Na: Sodium. [file 13048_2015_140_MOESM4_ESM.tiff]

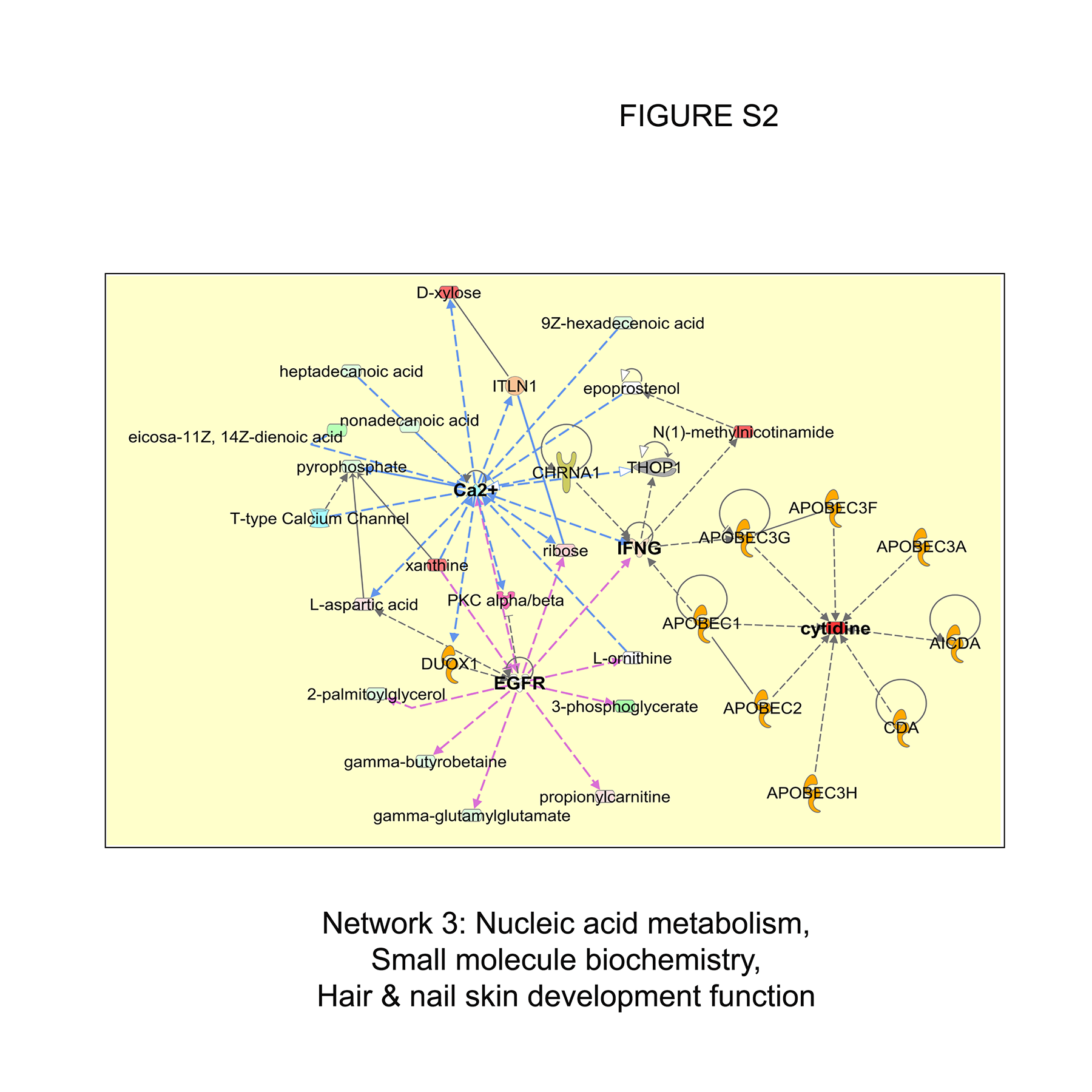

Supplement: Additional file 5: Figure S2. — Ingenuity Pathway Analysis (IPA) network 3. The third network contains components of nucleic acid metabolism and small molecule biochemistry. Constructed from the IPA knowledgebase using altered molecules, these networks are not limited by canonical pathway boundaries. Abbreviations: ITLN1: interlectin 1; CHRNA1: cholinergic receptor, nicotin alpha 1; Ca: calcium; PKC: protein kinase C; DuoX1: dual oxidase 1; EGFR: epidermal growth factor receptor 1; IFNG: interferon gamma; THOP1: human endopeptidase 1; APOBEC: DNA cytosine deaminase; AICDA: activation induced cytidine deaminase; CDA: cytidine deaminase. [file 13048_2015_140_MOESM5_ESM.tiff]

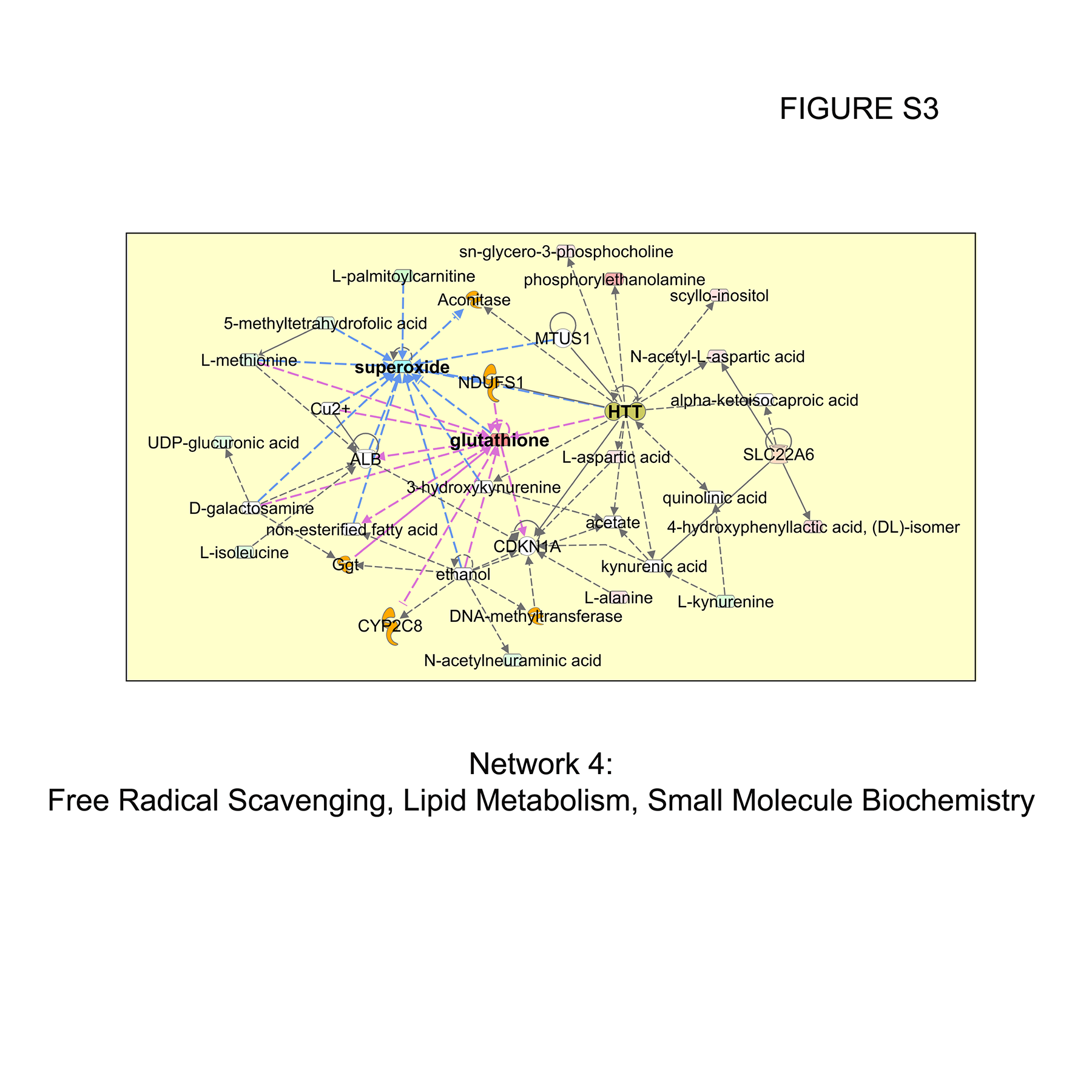

Supplement: Additional file 6: Figure S3. — Ingenuity Pathway Analysis (IPA) network 4. The fourth network contains components of free radical scavenging, lipid metabolism and small molecule biochemistry. Constructed from the IPA knowledgebase using altered molecules, these networks are not limited by canonical pathway boundaries. Abbreviations: Cu: copper; UDP: uridine diphosphate; MTUS1: mitochondrial tumor suppressor 1; NDUFS1: NADH-ubiquinone oxidoreductase iron-sulfur protein 1; CDKN1A: cyclin dependent kinase inhibitor 1A; CYP2C8: cytochrome 2C8; SLC22A6: solute carrier family 22 member 6. [file 13048_2015_140_MOESM6_ESM.tiff]

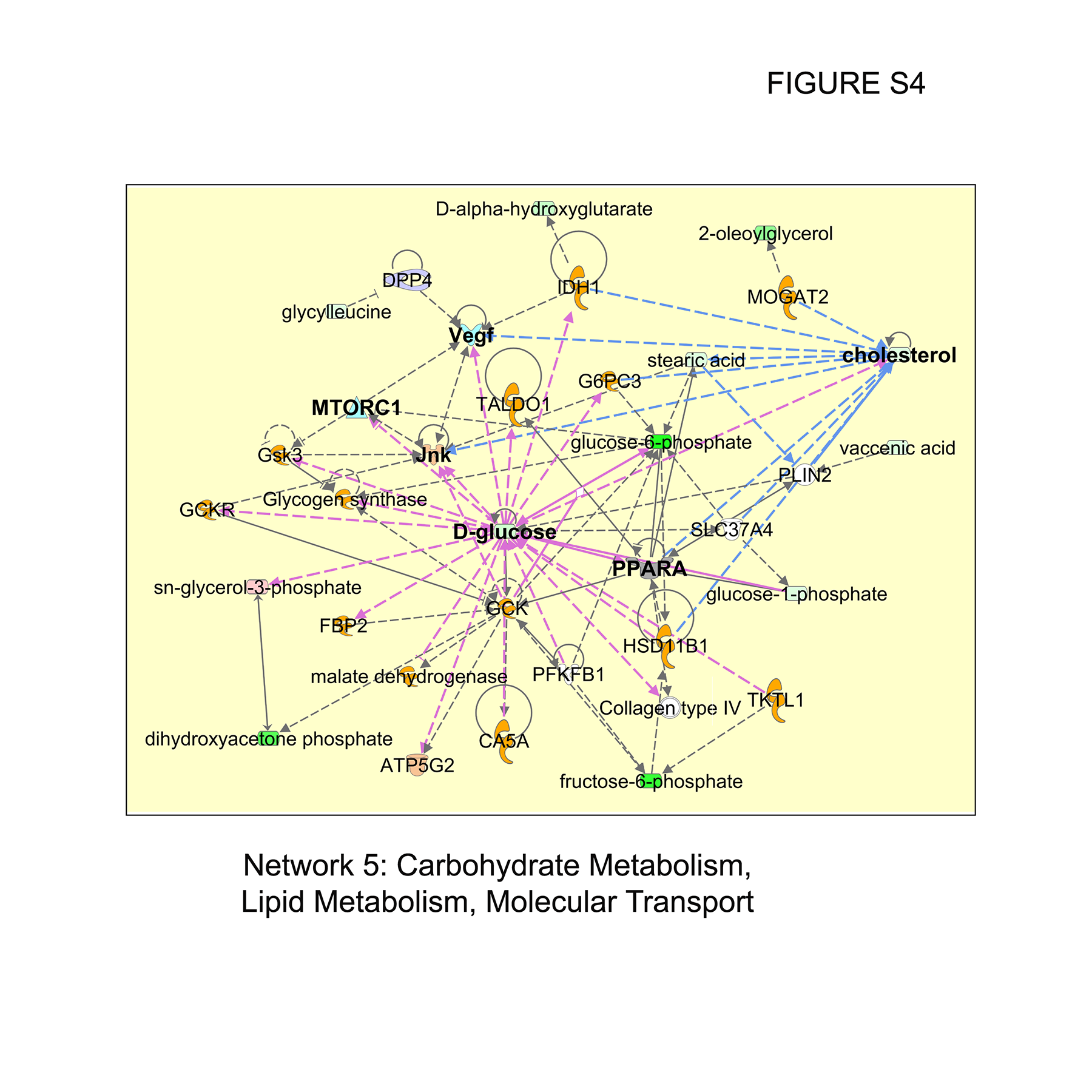

Supplement: Additional file 7: Figure S4. — Ingenuity Pathway Analysis (IPA) network 5. The fifth network contains components of carbohydrate metabolism, lipid metabolism and molecular transport. Constructed from the IPA knowledgebase using altered molecules, these networks are not limited by canonical pathway boundaries. Abbreviations: DPP4: dipeptidyl peptidase 4; VEGF: vascular endothelial growth factor; MTORC1: mammalian target of rapamycin complex1; JNK: c-jun N-terminal kinase; PPARA: peroxisome proliferator-activated receptor alpha; IDH1: isocitrate dehydrogenase 1; MOGAT2:acyl CoA: monoacylglycerol acyltransferase; TALDO1: transaldolase1; FBP2: phosphofruckokinase 2; CCK: cholecystokinin; PFKFB1: 6-phosphofructo-2-kinase/fructose-2,6-biphosphatase 4; HSD11B1: 11-beta-hydroxysteroid dehydrogenase type 1; TKTL1: transketolase 1; ATP5G2: ATP synthase; PL1N2: perilipin 2; CA5A: carbonic anhydrase VA. [file 13048_2015_140_MOESM7_ESM.tiff]
